# Gills as morphological biomarkers in extensive and intensive rainbow trout (*Oncorhynchus mykiss*, Walbaum 1792) production technologies

**DOI:** 10.1007/s10661-017-6278-7

**Published:** 2017-11-06

**Authors:** Emilia Strzyżewska-Worotyńska, Józef Szarek, Izabella Babińska, Dominika Gulda

**Affiliations:** 1 Department of Pathophysiology, Forensic Veterinary Medicine and Administration, University of Warmia and Mazury in Olsztyn, Oczapowskiego St. 13, 10-719 Olsztyn, Poland; 2 Department of Sheep, Goat and Fur Bearing Animal Breeding, University of Science and Technology, Mazowiecka St. 28, 85-084 Bydgoszcz, Poland

**Keywords:** Biomarker, Gills, Morphological lesions, Rainbow trout, Fish production technology

## Abstract

We investigated environmental impacts on rainbow trout (*Oncorhynchus mykiss*) reared at fish farms with either extensive technology, in a flow-through system (FTS, *n* = 3), or intensive technology, in a recirculating aquaculture system (RAS, *n* = 3). All fish were fed the same rations. Fish were caught in spring and autumn (body mass, 501–750 g) from these six farms. We performed macroscopic (intact fish) and microscopic (gills stained with haematoxylin/eosin) examinations. Lesions were categorised based on the type and location of structural abnormalities. The histopathological index (HAI) was calculated, and each lesion was scored. Fish reared in FTS or RAS were compared for the prevalence of morphological lesions. Gill epithelial hypertrophy and hyperplasia comprised 73% (RAS) to 79% (FTS) of all morphological abnormalities. In spring and autumn, lesions comprised, respectively, 11 and 18% (FTS) and 16 and 10% (RAS) mucous and chloride cell abnormalities and 8 and 4% (FTS) and 10 and 3% (RAS) blood vessel abnormalities. Diffuse, irreversible gill lesions were observed sporadically in all fish. Gill epithelium received the most exposure to environmental pathogens. HAIs indicated that normal gill architecture and minor lesions predominated in all fish. However, among trout caught in spring, moderate and extensive changes in gills occurred more commonly with RAS (34%) than with FTS (17%). Trout caught in autumn displayed no great differences. These results indicated that FTS prepared fish better than RAS for wintering. Moreover, we showed that gills were an excellent biomarker for analysing the impact of extensive and intensive production environments on rainbow trout.

## Introduction

When applied in morphological investigations, biomarkers are sensitive tools for the early detection of environmental changes (De la Torre et al. 2005; Heier et al. 2009; Marcovecchio 2004; Rayment and Barry 2000). Biomarkers demonstrate correlations between environmental factors and their outcomes, and they provide insight into both the status of an ecosystem and the status of a given organism (Flores-Lopes and Thomaz 2011; Fontainhas-Fernandes et al. 2008; Gernhofer et al. 2001; Maria et al. 2009; Sorour and Harbey 2012; Strzyżewska et al. 2015; Velcheva 2002; Yancheva et al. 2015). Fish gills comprise one of these biomarkers: the gill is a metabolically active tissue involved in gaseous exchange, and it accumulates a significant proportion of toxins (Abrahamson et al. 2008; Andres et al. 2000; Benli and Ozkul 2008; Evans et al. 2005; Marcovecchio 2004; Poleksic and Mitrovic-Tutundzic 1994; Strzyżewska et al. 2016; Thophon et al. 2003). Morphological lesions in the gills are easier to detect than functional abnormalities (Fanta et al. 2003; Fernandes et al. 2007; Velasco-Santamarίa and Cruz-Casallas 2008), because they may become visible long before behavioural changes in fish can be detected (Yancheva et al. 2015; Heier et al. 2009). Furthermore, the gills serve as early warning signs on the health status of fish (Sorour 2001). It is believed that the degree of morphological lesions in the gills can delimit the degree of environmental pollution (Camargo and Martinez 2007; Flores-Lopes and Thomaz 2011; Haaparanta et al. 1997; Ogundiran et al. 2009; Thophon et al. 2003). Indeed, the concentration of toxic factors in this organ is identical to the level of xenobiotics found in the water that the fish inhabit. In contrast, toxin concentrations in the other organs (such as the liver or kidneys) depict only the degree of distribution of the toxic compounds (Andres et al. 2000; Kroglund et al. 2008; Manera et al. 2016).

Importantly, fish are exposed both to environmental factors and to stress that results from manipulations, bioengineering and hydrotechnical procedures that occur during their production cycle (Cruz-Casallas et al. 2011; Tello et al. 2010; Yancheva et al. 2015). Current literature does not include any data from studies that examined whether the gills may serve as a morphological biomarker for evaluating fish production technologies. This study aimed to decrease this gap, because they discuss the pathomorphological aspects of rainbow trout gills that are modulated by common production systems, including the extensive flow-through system (FTS) and the intensive recirculating aquaculture system (RAS).

## Materials and methods

This study was carried out over two seasons (autumn and spring) and included six rainbow trout fish farms. The farms were selected with the following criteria: they had the same fish food rations and similar production volumes, marked-ready weight, and regional locations. Three of the farms operated with an extensive FTS, and the other three operated with an intensive RAS. Fish of 501–850 g (*n* = 6/farm) were collected and immediately sedated with 2-phenoxyethanol at a dose of 0.6–1.0 mL/dm^3^. Fish were then weighed and examined macroscopically. Specimens for microscopic investigations were sampled within 1–2 min after the fish were sacrificed. Specimens were acquired from the second gill arch situated on the right side of the head (Movahedinia et al. 2009). Tissue samples were immersed in fluids, fixed in 7% buffered formalin for 24 h, and then decalcified for 5–7 days in an EDTA solution (Flores-Lopes and Thomaz 2011). The tissues were fixed (Leica TP1020, Germany) and embedded to the long filament axis in paraffin blocks, i.e. two blocks for each collected fish (Leica EG1150H, Germany). The blocks were then cut into 5-μm sections, with a rotational Leica RM microtome (Germany). The preparations were stained with haematoxylin and eosin in a Leica Autostainex XL apparatus (Germany) (Bancroft and Gamble 2008). Two pathologists carried out microscopic examinations of 500 secondary lamellae in each preparation. The morphological lesions were photographed with an Eclipse 80i optical microscope, equipped with a Nikon PS-Fi1 digital camera and the NIS-Elements BR 2.30 program (Nikon, Japan).

Microscopic examinations considered the type and location of structural abnormalities. Lesions were categorised into four groups (G1–G4; Table 1). The categorised lesions were semi-quantitatively analysed, based on the severity of tissue damage. The first-stage lesions did not affect the normal functions of the tissue; the second-stage lesions were more advanced and had some impact on the functions of the tissue; the third-stage lesions were severe and caused irreversible damage to the organ. On the microscopic level, we implemented the histopathological index (HAI, as modified by Poleksic and Mitrovic-Tutundzic 1994). The HAI was calculated for each fish with the formula HAI = 1 × DI + 10 × DII + 100 × DIII, where DI, DII and DIII corresponded to the number of lesions classified as stage 1–3 degrees of severity, respectively, according to the score scale developed by Poleksic and Mitrovic-Tutundzic (1994). Moreover, each lesion was assigned a numerical scale from 0 to 6 to describe the extent, where 0 = no lesions, 2 = minor lesions, 4 = moderate lesions and 6 = extensive (diffuse) lesions (Bernet et al. 1999). We performed an analysis with the Statistica 10 StatSoft package to determine the location and variation measures for the variables examined for lesions in the G1–G4 groups. We compared the prevalence of gill lesions in trout reared with different production technologies (FTS and RAS), and statistical differences were calculated with a Mann-Witney *U* test and Bonferroni’s adjustment (StatSoft, Inc. 2013).

**Table 1:** Classification of microscopic lesions in the gills of rainbow trout, according to Bernet et al. (1999), modified for this study

Groups of microscopic lesions and types of lesion	Degree
G1: hypertrophy and hyperplasia of gill epithelia	
1. Hyperplasia of the gill filament epithelium	I
2. Hyperplasia of the lamellar epithelia	I
3. Decrease in interlamellar space	I
4. Epithelial lifting of gill filament epithelium	I
5. Epithelial lifting of lamellae	I
6. Intercellular oedema	I
7. Incomplete fusion of several lamellae	I
8. Complete fusion of several lamellae	I
9. Complete fusion of all lamellae	II
10. Rupture and peeling of gill filament epithelium	II
11. Rupture of the lamellar epithelium	II
G2: changes in mucous and chloride cells	
1. Hypertrophy and hyperplasia of mucous cells	I
2. Empty mucous cells or their disappearance	I
3. Hypertrophy and hyperplasia of chloride cells	I
G3: blood vessel changes	
1. Lamellar telangiectasis	I
2. Filament blood vessel enlargement	I
3. Haemorrhages with an epithelium rupture	II
4. Aneurysms	II
G4: terminal stages	
1. Fibrosis	III
2. Necrosis	III

## Results

We found that the gross appearance of rainbow trout was normal in most cases. Occasionally, some scale defects were apparent, and these were slightly more common in fish raised in a RAS than in those raised in a FTS. Hyperaemia in the liver was observed in 5 fish out of 72 and petechial was observed in 3 fish. These disturbances were reposted more often in spring, in the FTS group and in autumn in the RAS group.

**Fig. 1:**
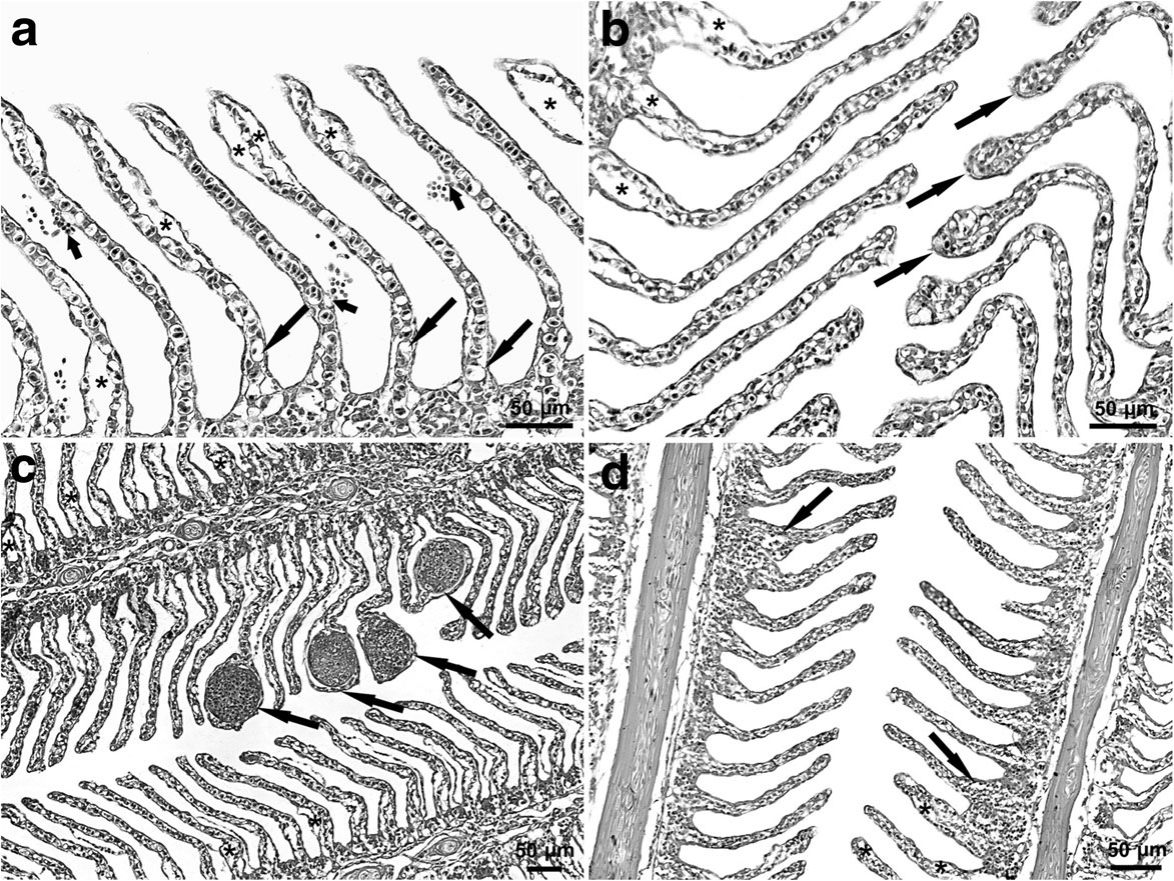
Morphological lesions in the gills of rainbow trout caught in spring. Micrographs show gill sections from fish reared with **a**, **b** extensive or **c**, **d** intensive technologies. (**a**–**d**) Epithelial lifting of varied severity (asterisks). **a** Haemorrhages (short arrows) and hypertrophy of mucous cells (long arrows). **b** Lamellar telangiectasis (arrows). **c** Lamellar aneurysms (arrows). **d** Filament hyperplasia with lamellar fusion (arrows). Most lamellae are intact on the left side. H&E staining. Scale bars: 50 μm

**Fig. 2:**
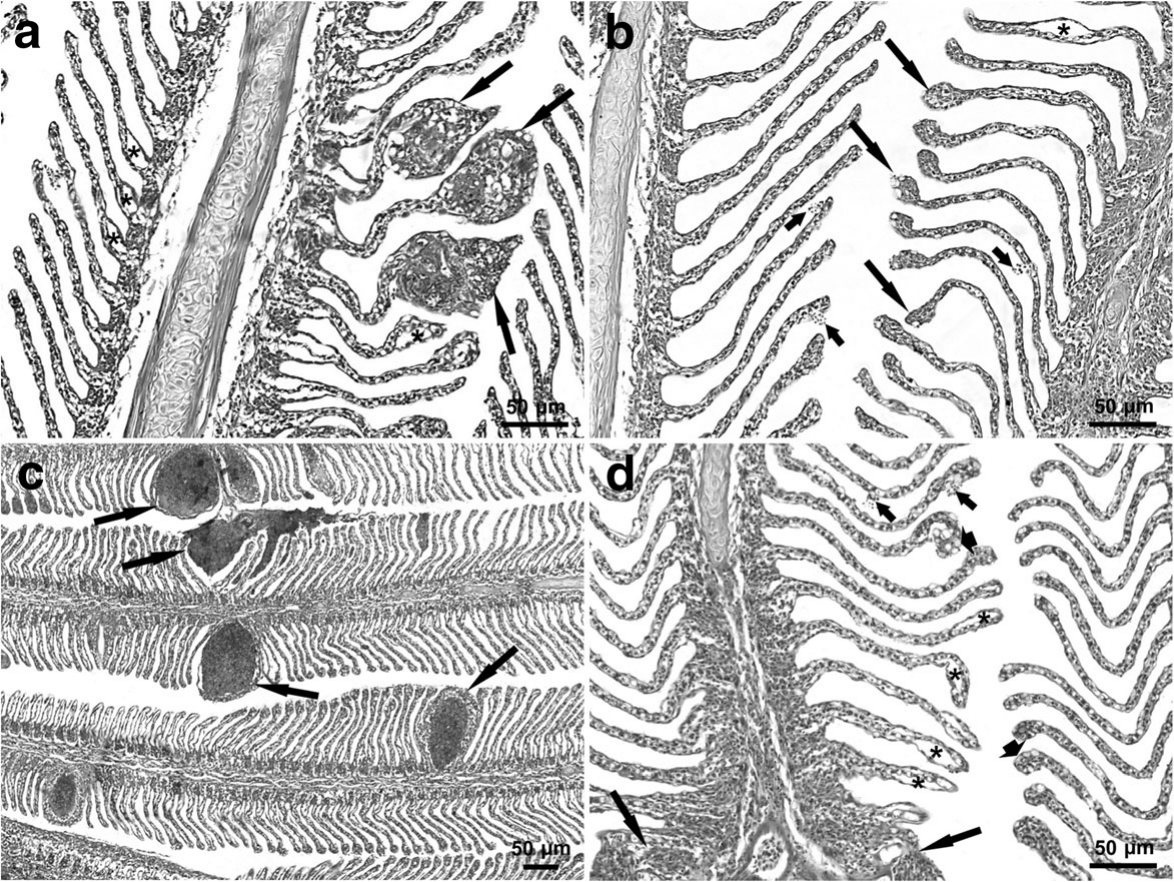
Morphological lesions in the gills of rainbow trout caught in autumn. Micrographs show gills from fish reared with **a**, **b** extensive and **c**, **d** intensive technologies. **a**, **b**, **d** Epithelial lifting of varied severity (asterisks). **a** Lamellar aneurysms (arrows). **b** Lamellar telangiectasis (long arrows) and haemorrhages (short arrows). **c** Lamellar aneurysms (arrows). **d** Filament hyperplasia with lamellar fusion (long arrows), lamellar telangiectasis (arrowheads) and haemorrhages (short arrows). H&E staining. Scale bars: 50 μm

The majority of rainbow trout had gills that displayed a normal microscopic pattern. All morphological lesions in this organ were detected microscopically, and they were present in both the spring and autumn, in both the FTS and RAS groups (Figs. 1 and 2).

**Fig. 3:**
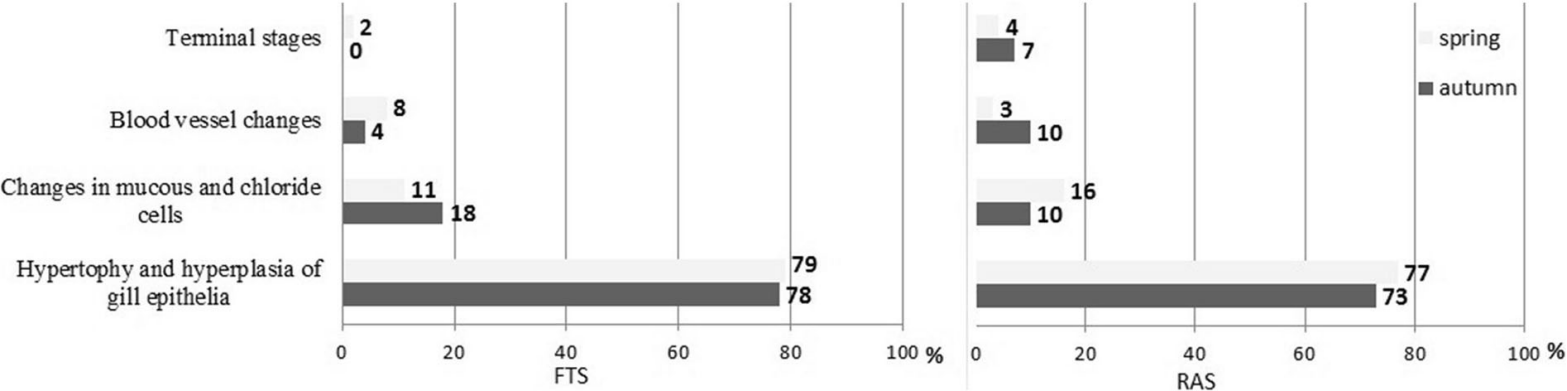
Prevalence (*x*-axes) of morphological lesions found on the gills of rainbow trout. Trout raised with the flow-through system (FTS) (*left*). Trout raised with the recirculating aquaculture system (RAS) (*right*). Shading indicates fish caught in the spring (light grey) and autumn (dark grey)

**Fig. 4:**
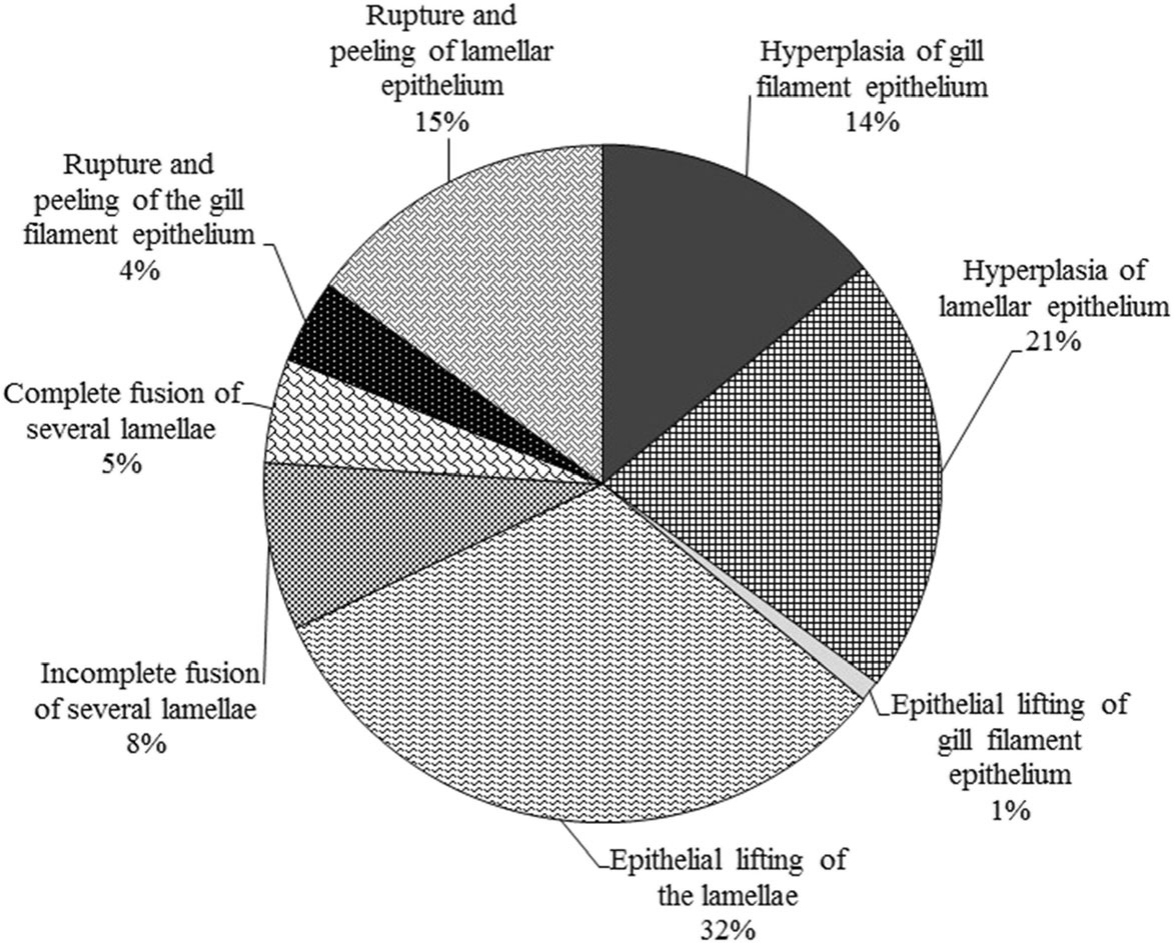
Prevalence of different types of epithelial hypertrophy and hyperplasia in gills of rainbow trout. Prevalence was measured in all trout (*n* = 72) raised in both the flow-through system (FTS) and the recirculating aquaculture system (RAS) and caught in both the spring and autumn periods

G1-type microscopic lesions in the gills constituted 73–79% of all detected abnormalities (Fig. 2). Gill epithelial lesions were most prevalent, followed by epithelial lifting of the lamellae and hyperplasia of lamellar epithelium (Fig. 3). Moreover, rupture and peeling of the lamellar epithelium and hyperplasia of gill filament epithelium were quite common. Incomplete and complete fusion of several lamellae and rupture and peeling of the gill filament epithelium were also reported (Fig. 4).

G2-type microscopic lesions, which involved mucous and chloride cells, constituted 10 to 18% of all observed morphological changes (Fig. 3). Hypertrophy and hyperplasia of mucous and chloride cells were observed more frequently in autumn than in spring in the FTS group, but they were more common in spring than in autumn in the RAS group.

G3-type abnormalities, which involved blood vessel changes, comprised 10% of all recorded microscopic lesions (Fig. 2). Among the vascular lesions, 46% were lamellar telangiectasia, 25% were aneurysms and 21% were haemorrhages due to epithelial rupture. Filament blood vessel enlargement was observed least frequently (8%). Blood vessel wall lesions in trout gills were observed more often in spring than in autumn in the FTS group, but more often in autumn than in spring in the RAS group.

G4-type lesions were rare; they constituted 0 to 7% of all observed microscopic abnormalities. These lesions were more often found in the RAS group than in the FTS group (Fig. 2).

The HAI values of fish were grouped into four ranges, representing low (0–10) to high (51–100) damage (Table 2). The fish caught in spring exhibited the entire range of HAI values (HAIs, 0–100). However, the fish caught in autumn exhibited only the two lowest HAI ranges (HAIs, 0–20).

**Table 2:** The percentage of rainbow trout within the indicated ranges of histopathological index values (HAIs, as modified by Poleksic and Mitrovic-Tutundzic 1994). HAI ranges indicate different degrees of damage on gills. Fish were caught in spring and autumn and reared in the flow-through system (FTS) or the recirculating aquaculture system (RAS)

HAI value range	Distribution of histopathological indexes (% fish)			
	FTS		RAS	
	Spring	Autumn	Spring	Autumn
0–10	72	50	22	33
11–20	11	50	44	67
21–50	6	0	28	0
51–100	11	0	6	0

**Fig. 5:**
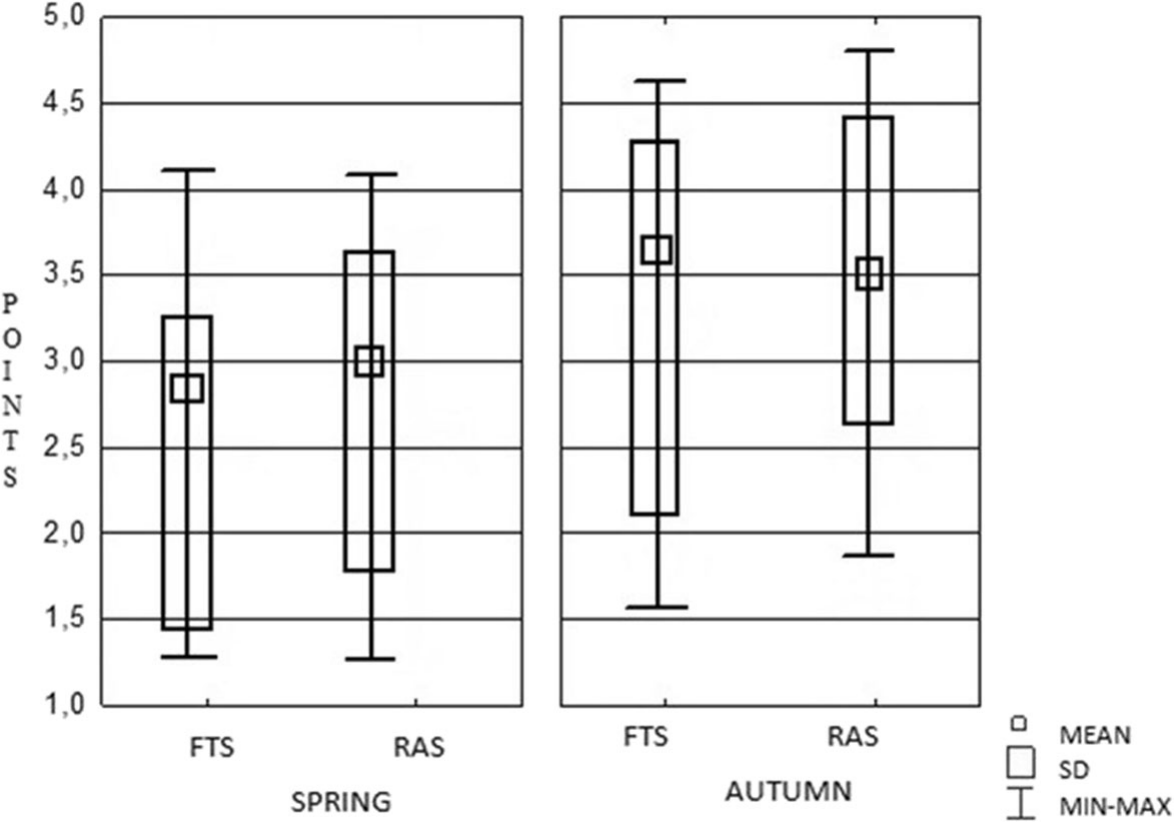
Distribution of scores (the point ranges from 0 to 6—Bernet et al. 1999) show the extent of damage observed in rainbow trout microscopic lesions. Trout were reared in the flow-through system (FTS) or recirculating aquaculture system (RAS), and they were caught either in spring (*left*) or autumn (*right*)

We also found that the FTS and RAS groups had significantly different average scores for the following descriptors: G1 DI, G2 DI and G3 DI (Table 3). The distribution of extreme values was significant for both the RAS and FTS technologies; that is, higher scores were recorded in autumn than in spring for both investigated systems. The FTS population demonstrated the highest individual variability. Furthermore, in autumn, the FTS group displayed significantly higher scores than the RAS group (Fig. 5).

**Table 3:** The average intensity scores for all morphological lesions on the gills of rainbow trout. The average score values (range 0–6) indicate intensity of morphological lesions, as detailed by Bernet et al. (1999). Lesions are grouped according to the microscopic stages (G) and degrees (D) of damage (Poleksic and Mitrovic-Tutundzic 1994). The analysis includes all 72 trout reared with the extensive (FTS) and intensive (RAS) technologies and caught in spring and autumn. Significance was based on the Mann-Whitney *U* test and Bonferroni’s adjustment (StatSoft, Inc. 2013)

Extent of morphological lesions in gills								
Group	Degree	FTS			RAS			Statistical significance
		n	Average score value	SD	n	Average score value	SD	
G1	DI	235	2.63	1.26	207	2.83	1.18	*
	DII	150	3.00	0.89	66	3.15	1.46	
	DIII	–	–	–	–	–	–	
G2	DI	16	2.19	0.91	16	2.56	0.96	*
	DII	–	–	–	–	–	–	
	DIII	–	–	–	–	–	–	
G3	DI	9	2.00	0.71	3	2.67	1.53	*
	DII	15	1.73	0.88	7	2.00	0.00	
	DIII	–	–	–	–	–	–	
G4	DI	–	–	–	–	–	–	
	DII	–	–	–	–	–	–	
	DIII	2	4.00	0.00	6	3.00	1.79	

*G1* hypertrophy and hyperplasia of gill epithelia, *G2* changes in mucous and chloride cells, *G3* blood vessel changes, *G4* terminal stages, *DI* first degree lesions that do not disturb normal tissue functions, *DII* second degree lesions, which are more advanced and impact tissue functions, *DIII* third degree lesions, which are severe and cause irreversible damage to tissues, *n* number of samples, *SD* standard deviation

*p ≤ 0.05

## Discussion

Fish gills have a thin, extensive surface that directly contacts the water, which makes this organ an entry point for multiple environmental factors (Baskar 2014; Haaparanta et al. 1997; Poleksic and Mitrovic-Tutundzic 1994). In addition, the gills are the primary sites for responding to unfavourable environmental changes (Benli and Ozkul 2008). These features imply that the gill epithelium receives the highest exposure to environmental pathogens. Our results have demonstrated that, among the observed microscopic lesions, the most common abnormalities involved epithelial cells, in both technologies and in both seasons (spring and autumn). Moreover, other researchers have described the lifting of fish gill epithelial cells as an outcome of exposure to metals (Nwani et al. 2010), organic pollutants (Fanta et al. 2003) and acute exposure to insecticides (Cengiz 2006). When these lesions, though non-specific, are evaluated at specific time intervals, changes in severity may indicate changes in the status of environmental quality.

Considering the mechanisms of action of pathogens, some authors have divided morphological changes into two types: lesions that result from direct damage by irritating factors and lesions that result from fish defence mechanisms (Baskar 2014; Kasherwani et al. 2009; Poleksic and Mitrovic-Tutundzic 1994). Based on findings from Velasco-Santamarίa and Cruz-Casallas (2008), it can be assumed that epithelial lifting, epithelial cell hyperplasia and hypertrophy, and partial adhesions of the gill lamellae are examples of fish defence mechanisms. These lesions result in an increased distance between the environment and the blood, and thus, they create an additional barrier for environmental factors (Baskar 2014; Poleksic and Mitrovic-Tutundzic 1994). Consequently, the epithelial surface available for diffusion is increased, and contaminant absorption into the blood is reduced. This defensive mechanism is triggered in fish in response to toxins, irritants and factors that induce environmental stress (Baskar 2014; Wendelaar Bonga 1997).

Chloride cell proliferation may be an adaptive process that responds to changes in the ionic balance of the environment (Mc Donald et al. 1991). Mucous cell stimulation is also an adaptive mechanism, which increases the amount of mucus secreted; an augmented mucus layer provides more effective protection to epithelial cells against environmental agents (Biagini et al. 2009; Paulino et al. 2014; Shephard 1994). In the present study, these cellular lesions were observed slightly more often in the FTS group than in the RAS group, throughout the year. Interestingly, these lesions were recorded 44% more often in the FTS group than in the RAS group in spring, but they were recorded 31% more often in the RAS group than in the FTS group in autumn. This demonstrated the variable impact that the environment had on trout reared with these two different production technologies.

Aneurysm formation is associated with pillar cell ruptures. Ruptures result from increased blood flow or from direct impact of contaminants on cells at a particular site. In the first stage, marginal channels become dilated, and the blood begins to congest in the gill lamellae (telangiectasia); in the terminal stage, aneurysms develop (Camargo and Martinez 2007; Paulino et al. 2014). Circulatory changes may be reversible, but they are far more difficult to reverse than epithelial changes (Poleksic and Mitrovic-Tutundzic 1994). In the present study, vascular lesions were found in a similar number of fish in both groups (Fig. 3), but they were slightly more common in the FTS group than in the RAS group in spring, and conversely, they were more common in the RAS group than in the FTS group in autumn. The comparison of aneurysms indicated that they were larger and more numerous per individual in the RAS than in the FTS group (Fig. 2). The results indicated that RAS trout had higher susceptibility to aneurysm formation than the FTS trout. This finding suggested that fish experienced worse environmental conditions in the intensive production system. In addition, our findings indicated that both rearing technologies induced seasonal susceptibility to aneurysms.

Severe lesions (GIII) that resulted in irreversible damage to the gills were relatively uncommon. Nevertheless, they occurred five times more frequently with the intensive technology (RAS) than with the extensive system (FTS; Fig. 3). A similar, but less pronounced finding was reported in another study, where single hepatocyte necrosis was strongly correlated to RAS technology than to FTS technology (Szarek et al. 2016).

We found that the HAI data was helpful for addressing the question of which technology created a more favourable environment for rainbow trout production. The HAI demonstrated that fish in the RAS group had twice as many (34%) moderate and extensive lesions than fish in the FTS group (17%). Importantly, this difference was only observed in fish caught in spring. The trout collected in the autumn did not display those types of lesions. In the autumn, HAI values for the FTS group indicated that organs functioned correctly, or only had minor damage (50% function for each situation). Similarly, the HAI scores for the RAS group indicated mostly (67%) minor gill damage or correct organ function. These findings showed that the environment was more favourable in summer than in winter; thus, trout reared with the FTS were better prepared for wintering than trout reared with the RAS.

It is important to keep in mind that lesions that affect gill morphology may cause disturbances in basic physiological processes, such as osmoregulation or antioxidative defence mechanisms. These disturbances take place much earlier in fish than changes in behaviour or exterior appearance (Yancheva et al. 2015). The results of this study and the multimodal analysis demonstrated that gills can serve as an excellent biomarker for analysing the impact of extensive and intensive production technology environments on rainbow trout.
